# ParticipACTION: A mass media campaign targeting parents of inactive children; knowledge, saliency, and trialing behaviours

**DOI:** 10.1186/1479-5868-6-88

**Published:** 2009-12-09

**Authors:** Cora L Craig, Adrian Bauman, Lise Gauvin, Jennifer Robertson, Kelly Murumets

**Affiliations:** 1 Canadian Fitness and Lifestyle Research Institute, Ottawa, Canada; 2 School of Public Health, University of Sydney, Sydney, Australia; 3 Department of Social and Preventive Medicine, Centre de Recherche Lea-Roback sur les Inégalités Sociales de Santé de Montréal, Université de Montréal, Montréal, Canada; 4 Centre de recherche du Centre Hospitalier de l'Université de Montréal (CRCHUM), Montréal, Canada; 5 ParticipACTION, Toronto, Canada

## Abstract

**Background:**

In late 2007, Canada's ParticipACTION national physical activity mass media campaign was re-launched, with an initial campaign targeting parents of elementary school-aged children. The campaign informed them about the risks of physical inactivity for children and youth. The purpose of this study was to assess campaign awareness and understanding following the campaign, and to identify whether exposure to this campaign was likely associated with behaviour change.

**Methods:**

A convenience sample of 1,500 adults was recruited though an existing panel (n = 60,000) of Canadian adults to participate in online surveys. Initial campaign exposure included "prompted" and "unprompted" recall of specific physical activity messages from the 2007 ParticipACTION campaign, knowledge of the benefits of PA, saliency, and initial trial behaviours to help their children become more active.

**Results:**

One quarter of respondents showed unprompted recall of specific message content from the ParticipACTION campaign, and prompted recall was 57%. Message recall and understanding was associated with knowledge about physical activity, and that in turn was related to high saliency. Saliency was associated with each of the physical activity-related trial behaviours asked.

**Conclusion:**

Campaign awareness and understanding was high following this ParticipACTION campaign, and was associated with intermediate campaign outcomes, including saliency and trial behaviours. This is relevant to campaign evaluations, as it suggests that an initial focus on influencing awareness and understanding is likely to lead to more substantial change in campaign endpoints.

## Background

Communication campaigns are an important public health strategy to encourage individuals to become more active, yet few are evaluated. For example, in Canada, there was a long tradition of mass media campaigns through ParticipACTION (1973-2001) but limited assessment of their effectiveness, apart from sustained high-level brand awareness, with serial studies observing ParticipACTION brand recognition in over three quarters of the population [1,2]. One model that is commonly used to design campaign messages is the Hierarchy-of-Effects model [3]. Briefly, this model proposes that media campaigns might work by influencing initial awareness (proximal variables) through intermediate impact on knowledge, saliency, attitudes/health beliefs, self efficacy, and intention [4] to longer-term effects (distal variables), which include the trialing and adoption of physical activity (PA) behaviours. This model informed the design of ParticipACTION's campaign, which re-launched in October 2007. The campaign messages were designed to influence knowledge of the health consequences of physical inactivity among children, and to make this a salient issue among parents of 7 to 12 year olds and among adults more generally. This campaign was intended to initiate changes in awareness, and lay the foundation for subsequent campaigns. As with previous campaigns and theoretical frameworks for mass media, this ParticipACTION campaign focused on impacting message awareness, understanding (knowledge), and recognition of the issue (saliency), as these are considered the most important initial outcomes for mass-reach campaigns [5,6].

A total of seven messages (four English, three French) were aired on 8,390 television spots in English and 573 television spots in French from early morning to late evening on major Canadian networks (e.g., CTV, the Canadian Television Network) and specialty channels (e.g., HGTV, the Home and Garden Network). In total, 20 spots were aired on English-language stations and five on French-language stations, which is reflective of the proportion of French-speaking residents in Canada (22%) [7]. The total audience for all television spots was estimated at over 140 million for an average of 5.4 exposures among Canadians aged 15 years and older.

This paper reports on the short-term post-campaign measures of campaign exposure, and their association with knowledge and trialing of physical activity-related behaviour. Recent evidence from the VERB mass media US-wide campaign demonstrated that for young people aged 9 to 13 years, knowledge or understanding of the campaign's physical activity messages was a critical proximal variable that was a useful indicator of subsequent campaign impact [8].

## Methods

### Respondent recruitment

The investigation was an initial part of ongoing evaluation activities performed at ParticipACTION. In this initial post-campaign survey, respondents were recruited from an existing online panel (n = 60,000) of Canadian adults accrued by Angus Reid Strategies, comprised of individuals who had given consent to be contacted for research. Canadians 18 years of age and older were randomly selected from a register that was stratified by region, age, and gender to reflect the demographic distribution of the overall Canadian population using Statistics Canada census data. Parents of children 7 to 12 years of age were oversampled for this study using demographic information provided when recruited to the panel, and then screened to ensure they had children from 7 to 12 years of age in the home. Respondents were sent an email inviting them to respond online to a survey, and willing respondents followed unique links provided in the emails to the internet-based survey instrument. The questionnaire took approximately 10 minutes to complete. Participants were not informed ahead of time about the specific content of the survey, but were advised that their responses would be kept strictly confidential, and a link to the company's privacy policy to protect confidentiality and privacy was provided. Surveys were completed from April 4^th ^to April 17^th^, 2008. Recruitment ceased once 1,500 people had completed the questionnaire. Participation in the study overall, and for any individual item, was completely voluntary although a small incentive ($3) was offered. A copy of the denominalized dataset was provided to ParticipACTION and thus participants were anonymous. Ethics approval for this study was obtained from the Human Research Ethics Committee of the Faculty of Medicine at the Université de Montréal.

### Measures

The immediate effect of the campaign was assessed through unprompted and prompted recall of this ParticipACTION campaign's mass media messages. Respondents were asked if they had seen any media messages within the past three months related to physical inactivity among children and, if they had, they were asked to specify the content of the messages. Multiple responses were permitted, which were coded across responses as "physical inactivity leads to increased health risks", "physical activity reduces risks", other specific messages consistent with ParticipACTION messages, responses not consistent with the campaign, "don't know", and "did not see any media messages". Subsequently, respondents were presented with text descriptions and several visual frames of the ParticipACTION messages appropriate to the language of completion. The number of prompted campaign messages was summed from the individual's prompted recall associated with each of the campaign's probes. Prompted recall was defined as having seen at least one of the messages presented through a website link. Other potential short-term effects that were measured included:

• knowledge of the risk of physical inactivity (i.e., "Physical inactivity leads to a host of chronic degenerative conditions and premature death" and "Today's children have a lower life expectancy than their parents"), which was measured using a four-point bi-polar agreement scale (strongly disagree to strongly agree). For each question, the two disagree categories were contrasted to the agree categories.

• saliency or personal relevance of the need to be physically active (i.e., "And, how concerned would you say you are personally about: (i) the issue of physical inactivity among children, (ii) the risks associated with physical inactivity for children, (iii) the issue of physical inactivity among all Canadians, and (iv) the risks associated with physical inactivity among all Canadians?"), which was measured employing a four-point scale from not at all concerned to very concerned. The four items were summed (Cronbach α = 0.90) and categorized as an approximate quartile split: very low (0-7), low (8-9), moderate (10-11), and high (12).

Initial physical activity-related trial behaviours were actions that adults took towards becoming more active; these were provided as a checklist to those recalling at least one message. Trialing behaviours were attributed to the messages, through the stem in the question: "As a result of seeing this particular message, have you done any of the following?" Parents received all of the following items whereas those without children living at home were provided with only the first three items: "Started doing more physical activity myself and/or as a family"; "Looked for information about physical activity"; "Visited the website for the organization that sponsored the ad"; "Made stricter rules for your child/children with regard to time spent on sedentary activities (e.g., watching TV, playing video games, etc.)"; "Talked to your child/children about being more active"; and "Talked to your spouse or partner about encouraging the child/children to be more active". Although it was possible to construct a moderately reliable overall score (Cronbach α = 0.72), we were interested in each type of trial behaviour and thus did not create a composite scale. As fewer than 3% of respondents "visited the website", these responses were not included in the analysis.

In addition, information was collected on the number of days in the previous week that the respondent participated in 60 or more minutes of physical activity, the number of days in the previous week that their children participated in 60 or more minutes of physical activity, and basic demographic information (age group, gender, education, income, and language in which the respondent chose to complete the survey).

### Analysis

Post-campaign recall (prompted and unprompted) was described, and the relationship of demographic factors to recall was examined using multiple logistic regression. The associations between recall, awareness, knowledge, salience, and behaviour were assessed separately among adults and among parents of elementary school-aged children using separate multiple logistic regressions for each behaviour, adjusting for age, sex, education, income, language, and activity level (adult and child). All analyses were performed using SPSS version 15. Models using forced entry and backward elimination of variables were examined, yielding similar results. Results for adjusted models using forced entry are reported.

### Results

Table 1 describes characteristics of individuals responding to the survey. Roughly half of respondents were parents of children between the ages of 7 and 12 years (n = 800) and the remainder were other parents and adults (n = 754). Overall, respondents differed from the Canadian population in that there were relatively more adults aged 35 to 54 years, with college or technical school education, and living in households earning $50,000 to $99,999 annually, and fewer aged 55 years or older, and who completed the questionnaire in French. In addition, relatively more parents of 7 to 12 year olds lived in high income households (≥ $100,000 annually), and more of the other adults were university graduates.

**Table 1:** Comparison of sample characteristics and with population statistics according to the 2006 Statistics Canada Census

	*2006 Census*		*Parents of 7-12 year olds* *(n = 800)*	*Other Adults* *(n = 754)*
	*%*	*n*	*% (95% CI)*	*% (95% CI)*
**Demographics**				
Sex				
Men	48.2	763	47.4 (43.9-50.8)	50.9 (47.4-54.5)
Women	51.8	791	52.6 (49.2-56.1)	49.1 (45.5-52.6)
				
Age				
18-34 years	28.0	294	16.9 (14.4-19.6)	21.1 (18.3-24.1)
35-54 years	39.7	1014	78.9 (75.9-81.6)	50.8 (47.2-54.4)
55 and older	32.2	246	4.3 (3.1-5.9)	28.1 (25.0-31.4)
				
Education				
HS or less	39.4	313	19.6 (17.0-22.5)	20.7 (17.9-23.7)
College/Tech School	32.7	762	50.1 (46.7-53.6)	47.9 (44.3-51.5)
University+	27.9	479	30.3 (27.2-33.5)	31.4 (28.2-34.8)
				
Income				
<$50000	46.6	461	28.4 (25.4-31.6)	31.0 (27.8-34.4)
$50000-$99000	34.0	614	41.4 (38.0-44.8)	37.5 (34.1-41.1)
≥ $100000	19.4	347	23.3 (20.4-26.3)	21.4 (18.6-24.4)
No Response	-	132	7.0 (5.4-9.0)	10.1 (8.1-12.4)
				
Language of survey				
English	58.0	1296	82.4 (79.6-84.9)	84.5 (81.7-86.9)
French	22.0	258	17.6 (15.1-20.4)	15.5 (13.1-18.3)

When asked if they had seen any messages related to physical inactivity among children in the media in the three months prior to the survey, 22.3% of respondents (95% CI: 20.3, 24.5) described a message consistent with the specific messaging of the ParticipACTION campaign without prompting: 14.9% (95% CI: 13.2, 16.7) indicated that the key message of the campaign was that physical inactivity is associated with increased risk of chronic conditions; 5.9% (95% CI: 4.8, 7.2) stated that physical activity was associated with health benefits; and the remaining 1.6% (95% CI: 1.0, 2.3) provided another description for the content of the message that was consistent with the message or image of the campaign (e.g., children talking about hip replacement). The remaining respondents had not seen any physical activity message (59.8%, 95% CI: 57.3, 62.2) or had seen other messages (17.9%, 95% CI: 16.1, 19.9) that were clearly not related to ParticipACTION (e.g., for a fitness club) in the previous three months.

Unprompted recall was more likely among women and those with a tertiary level of education and less likely among those responding in French; it also showed a trend towards increased likelihood of recall among high active adults and children (Table 2).

**Table 2:** Association of physical activity and demographic characteristics with unprompted and prompted recall of ParticipACTION campaign

		*Unprompted recall* *% (95% CI)*	*OR*_*adj *_*(95% CI)*	*Prompted recall* *% (95% CI)*	*OR*_*adj *_*(95% CI)*
**Physical activity and demographics**					
Sex	Men (referent)	19.7 (17.0-22.6)	1.00	52.3 (48.7-55.8)	1.00
	Women	25.2 (22.3-28.3)	1.4 (1.1-1.8)	61.2 (57.7-64.5)	1.4 (1.1-1.7)
Age group	18-34 years (referent)	24.5 (19.9-29.7)	1.00	59.2 (52.5-64.7)	1.00
	35-54 years	22.9 (20.4-25.6)	0.9 (0.7-1.2)	58.6 (55.5-61.6)	1.1 (0.8-1.4)
	55 and older	18.3 (13.9-23.6)	0.7 (0.5-1.2)	46.7 (40.6-53.0)	0.6 (0.4-0.9)
Population segment	Non-parent (referent)	18.7 (14.9-23.3)	1.00	54.4 (51.0-59.9)	1.00
	Parent of 7-12 year olds	23.0 (20.2-26.0)	1.2 (0.9-1.8)	58.5 (55.0-61.9)	1.0 (0.7-1.3)
	Parent of other-aged children	24.3 (20.5-28.7)	1.4 (1.0-2.1)	55.6 (50.8-60.2)	1.0 (0.7-1.3)
Education	HS or less (referent)	18.5 (14.5-24.3)	1.00	57.8 (52.3-63.2)	1.00
	College/Tech School	22.6 (19.7-25.7)	1.3 (0.9-1.8)	59.3 (55.8-62.8)	1.0 (0.8-1.4)
	University+	24.2 (20.6-28.3)	1.5 (1.0-2.2)#	52.2 (47.7-56.6)	0.8 (0.6-1.1)
Income ($1000s)	<50 (referent)	23.2 (19.6-27.3)	1.00	57.7 (53.1-62.1)	1.00
	50-99	21.7 (18.6-25.1)	0.9 (0.7-1.2)	56.8 (52.9-60.7)	1.0 (0.8-1.3)
	≥ 100	21.9 (17.9-26.6)	0.8 (0.6-1.2)	53.3 (48.0-58.5)	0.9 (0.7-1.2)
	DK/Ref	25.0 (18.3-33.1)	1.1 (0.7-1.7)	62.9 (54.3-70.7)	1.2 (0.8-1.9)
Language of survey	English (referent)	22.5 (21.2-25.8)	1.00	58.2 (54.5-60.8)	1.00
	French	17.4 (13.3-22.6)	0.7 (0.5-1.0)#	50.0 (43.9-56.1)	0.7 (0.5-0.9)
Adult's physical activity level	None (referent)	19.1 (15.3-23.6)	1.00	51.5 (47.2-57.7)	1.00
	1 day	23.6 (19.0-28.9)	1.3 (0.9-2.0)	58.6 (52.7-64.2)	1.3 (0.9-1.8)
	2-3 days	24.0 (20.5-27.8)	1.3 (1.0-1.9)	59.6 (55.4-63.7)	1.4 (1.0-1.8)
	4+ days	22.6 (18.7-26.9)	1.3 (0.9-1.8)	55.6 (50.7-60.4)	1.2 (0.8-1.5)
Child's physical activity level	None (referent)	17.3 (10.3-27.6)	1.00	44.0 (33.2-55.4)	1.00
	1 day	19.7 (13.5-27.7)	1.2 (0.6-2.6)	47.5 (38.8-56.4)	1.1 (0.6-2.1)
	2-3 days	22.6 (19.1-26.6)	1.4 (0.7-2.7)	57.1 (52.6-60.5)	1.6 (1.0-2.7)
	4+ days	25.9 (22.4-29.7)	1.6 (0.8-3.1)	61.8 (57.7-65.8)	1.9 (1.1-3.2)

Odds ratio for the full model for all respondents including age, sex, population segment, education, language, adult PA level, and for parent's and child's PA level.

# Lower 95% CI is 1.01

When prompted, 25.2% (95% CI: 23.1, 27.4) indicated that they had seen one message, 20.5% (95% CI: 18.5, 22.5) recalled seeing two, and 11.1% (95% CI: 9.7, 12.8) reported seeing three or more messages. As with unprompted recall, prompted recall was more likely among women and among parents of high active children, and was less likely among those completing the survey in French.

Overall, 58.8% (95% CI: 56.3, 61.2) stated that they strongly agreed that "physical inactivity is associated with higher risk of chronic health problems". Agreement was more likely among those who recalled the campaign message, those with higher educational attainment, Francophones, and those who were more active (data not shown). The second knowledge item (agreement that today's children might have a lower life expectancy than their parents) was not part of the campaign messaging and it had a lower prevalence of agreement (overall 12.4%; 95% CI: 10.8, 14.1) than the first knowledge item and was unrelated to unprompted and prompted recall for parents and for adults more generally.

Roughly equal proportions of respondents had moderate (24.1%, 95% CI: 22.1, 26.3) and high (27.5%, 95% CI: 25.4, 29.8) saliency scores. The remainder had low (35.0%, 95% CI: 32.7, 37.4) or very low (13.3%, 95% CI: 11.7, 15.1) scores on the saliency scale. Among adults with and without children alike, having a high saliency score was unrelated to either unprompted or prompted recall. High personal relevance of physical activity was more frequent among women, physically active adults, and was related to agreement with both knowledge items.

As a result of seeing the ParticipACTION commercials, 8.5% of respondents reported that they "looked for information about physical activity", 2.5% "visited the website for the organization that sponsored the message", and 26.5% reportedly "started doing more physical activity" as an individual or as a family. Among parents, 33.1% talked to their children about "being more active", 22.0% talked to their spouse or partner about "encouraging the child/children to be more active", 19.9% "made stricter rules" about time spent on sedentary activities, and 12.2% enrolled their children "in an organized physical activity or sport".

Although knowledge of the risks of physical inactivity were not associated with trialing behaviours, unprompted recall of the ParticipACTION campaign was associated with talking to one's spouse about their children's activity level and increasing physical activity among the respondent or their family. The number of prompted messages recalled was also related to talking to one's spouse about children's activity, increasing physical activity, and enrolling children in physical activities. High personal relevance of physical activity was significantly associated with each of the physical activity-related trial behaviours (Table 3). In addition, recalling at least three messages was associated with each of the initial trial behaviours investigated, with the exception of making stricter rules about sedentary activity (here, higher odds were associated with seeing two of the messages).

**Table 3:** Association between initial trialing behaviors and recall, knowledge of benefits of and saliency for physical activity among parents

	*Talked to their children about increasing PA* *OR*_*adj *_*(95% CI)*	*Talked to spouse about increasing child's PA* *OR*_*adj *_*(95% CI)*	*Made stricter rules about sedentary activity* *OR*_*adj *_*(95% CI)*	*Enrolled children in PA* *OR*_*adj *_*(95% CI)*	*Increased PA (own or as a family)* *OR*_*adj *_*(95% CI)*	*Sought information about PA* *OR*_*adj *_*(95% CI)*
**Recall, knowledge and saliency**						
Unprompted recall^a^						
Didn't recall	1.0	1.0	1.0	1.0	1.0	1.0
Recalled message	1.2 (0.9, 1.7)	1.6 (1.0, 2.3)#	1.2 (0.8, 1.8)	0.6 (0.4, 1.1)	1.7 (1.1, 2.4)	1.5 (0.9, 2.6)
Prompted recall^b^						
None						
1 ad	1.0	1.0	1.0	1.0	1.0	1.0
2 ads	1.0 (0.8, 1.6)	1.2 (0.8, 1.9)	1.6 (1.0, 2.5)#	1.3 (0.8, 2.3)	1.5 (1.0, 2.3)#	1.9 (0.9, 3.9)
3 or more ads	1.9 (1.2, 3.0)	1.7 (1.1. 2.8)	1.3 (0.7, 2.2)	2.0 (1.1, 3.7)	2.2 (1.4, 36)	3.3 (1.6, 6.9)
Physical inactivity increases risks						
Disagree	1.0	1.0	1.0	1.0	1.0	1.0
Agree	1.3 (0.5, 3.2)	0.8 (0.3, 2.3)	1.0 (0.3, 3.4)	0.7 (0.2, 2.3)	0.8 (0.3, 2.1)	0.6 (0.1, 3.0)
Strongly agree	1.6 (0.6, 4.1)	0.8 (0.3, 2.4)	1.2 (0.4, 3.7)	0.7 (0.2, 2.5)	0.9 (0.4, 2.3)	0.9 (0.2, 4.5)
Kids have lower life expectancy						
Disagree	1.0	10.	1.0	1.0	1.0	1.0
Agree	1.1 (0.7, 1.6)	1.0 (0.7, 1.6)	1.0 (0.7, 1.6)	1.2 (0.7, 2.1)	1.0 (0.6, 1.4)	1.6 (0.8, 3.0)
Strongly agree	0.8 (0.5, 1.4)	0.7 (0.4, 1.3)	1.2 (0.6, 2.1)	0.6 (0.2, 1.4)	0.9 (0.5, 1.6)	1.1 (0.5, 2.7)
Saliency						
Very low	1.0	1.0	1.0	1.0	1.0	1.0
Low	1.9 (1.0, 3.9)	5.8 (1.7, 19.7)	2.4 (0.9, 6.5)	2.7 (0.8, 9.7)	1.8 (0.9, 3.8)	1.1 (0.2, 5.5)
Moderate	4.2 (2.1, 8.7)	11.8 (3.4, 40.1)	5.2 (1.9, 14.0)	7.5 (2.2, 26.4)	3.6 (1.7, 7.5)	4.0 (0.9, 18.3)
High	5.9 (2.9, 12.2)	16.0 (4.7, 54.8)	8.1 (3.0, 21.6)	7.9 (2.3, 27.7)	4.7 (2.3, 9.9)	6.3 (1.4, 28.0)

^a ^Odds ratio for full model including unprompted recall adjusted for age, sex, education, income, language, adult PA level, and for parent's and child's PA level.

^b ^Odds ratio for full model including number of ads recalled adjusted for age, sex, education, income, language, adult PA level, and for parent's and child's PA level.

# Lower 95% CI is 1.01

## Discussion

The purpose of this paper was to report on the post-campaign awareness and actions taken by Canadian adults and parents of elementary school-aged children following the 2007-2008 ParticipACTION mass media campaign. We found 22% of respondents recalled a message strongly related to the ParticipACTION campaign without prompting and 57% recalled at least one of the specific messages when prompted. The latter compares favourably to the prompted recall associated with Canada on the Move [9], another recent Canadian physical activity campaign. In global terms, this level of unprompted and prompted awareness is above average for physical activity campaigns [5]. In this campaign, recognition of the ParticipACTION brand was even higher [2], but the present evaluation focused conservatively on more specific message recognition. Further, during this period there were no competing public youth-targeted physical activity mass-reach campaigns in Canada at the national level; however, lower recall rates were noted among respondents answering in French, possibly due to fewer messages being aired in the French language.

Message recall, as with other mass campaigns, was more frequent among women, tertiary-educated adults, and physically active adults. Recall was related to knowledge and to perceived relevance (saliency), which in turn was associated with several physical activity-related trial behaviours.

Although the campaign generally reached all segments of society, there were language, education, and adult activity level differentials in knowledge and in personal relevance of physical activity. This suggests that audience segmentation with messaging might be required to increase knowledge and personal relevance of physical activity among these groups. Since prompted recall and saliency were associated with most to all of the trial behaviours, targeting these initial components could be a useful communications strategy.

Twenty-year trends in physical activity from 1981 to 2000--a period during which earlier ParticipACTION campaigns were run--showed that women were less active than men, that gradients occurred by age and education, and that an income differential that was not apparent in 1981 had emerged by 2000 [10]. It therefore would be prudent to note that awareness of this ParticipACTION campaign was across diverse segments of the Canadian population. For example, although unprompted recall differed by education and there was a trend to higher awareness with parental activity level, unprompted recall did not differ by income level, by the physical activity level of children, by parental status, or between adults with and without children aged 7 to 12 years living at home. This suggests that the campaign may have reached parents with both more active and less active children and some segments of society more likely to be inactive (e.g., women, older adults).

There are a number of important limitations with the current study. First, the sample was drawn from a non-representative panel of individuals who previously had agreed to participate in surveys. A further limitation was the self-report nature of responses, especially regarding trialed behaviours. In addition, the survey design was cross-sectional, and carried out only following the ParticipACTION campaign. For recall questions, however, there were no competing mass campaigns and recall, especially unprompted, is likely to be real. Specific prompted recall questions were asked, in order to reduce the residual "generic ParticipACTION" recall, even seven years after the previous ParticipACTION campaign [11]. Personal relevance and current physical activity were also correlated with trialing physical activity-related behaviours, but this is not unexpected, as these are likely antecedents of behavioural trialing, although a longitudinal design would be essential to sequence these variables, and test the "cascade" or Hierarchy-of-Effects model [1,3,4]. Finally, we could not examine change in reported activity behaviours among children or their parents, but instead assessed reported trialing of antecedent behaviours that would lead to activity. Physical activity campaigns occasionally lead to increases in reported physical activity if the behaviour is specific and trialable [12], as occurred in the Canada on the Move campaign in 2004 [9,13], or if the mass media is sustained and extensive [14]. For most campaigns, however, these behavioural effects are often small [15-18].

## Conclusion

Mass media communication and social marketing campaigns are key ingredients in raising awareness of the issue of inactive lifestyles, and have been shown to be an effective component of public health strategies to increase physical activity [5-7,19]. This study shows high levels of post-campaign awareness of the ParticipACTION messages, which reached many population groups including parents of school-aged children. As future ParticipACTION campaigns roll out, a fuller examination of their impact on knowledge and saliency, and additional elements such as attitudes, self-efficacy, and future intention to be active, are required, using longitudinal designs. Nonetheless, the resurgence of ParticipACTION is associated with encouraging levels of community awareness and message reach, and a possible initial behavioural response.

## Competing interests

CLC and LG were members of the Content and Collaboration Committee of ParticipACTION. KM is President and Chief Executive Officer of ParticipACTION and JR is Knowledge Manager. AB declares no competing interests.

## Authors' contributions

CLC conceptualized the paper and was a significant manuscript writer. LG and AB contributed to the writing of the manuscript and conceptualization of the paper. JR led the design of the survey instrument and the data acquisition phase and reviewed the manuscript. KM was the lead in the conceptualization of the data collection study, and provided critical review of the manuscript.
